# Meteorological Table

**Published:** 1810-12

**Authors:** 


### METEOROLOGICAL TABLE.

### *From Oct. 29, to Nov. 27.*

| D | Therm. | Barom. | Hygrom. dry | damp. | Weather. |
|---|---|---|---|---|---|
| 29 | 70 43 36 | $29^{6}$ $29^{7}$ — | ———10 | ——— | clear . * ——— . . . . ——— . . . . |
| 30 | 37 42 36 | $29^{8}$ $29^{9}$ — | ——— | 27 — 15 | Snow . . clear . . . ——— . . . . . |
| 31 | 35 43 44 | 30 $29^{9}$ $29^{3}$ | ——— | 15 10 28 | fog . . cloudy . . rain . |
| 1 | 49 47 42 | $29^{7}$ $29^{6}$ $29^{7}$ | ——— | 27 22 — | cloudy . . rain . cloudy . . . |
| 2 | 41 47 42 | $29^{9}$ — — | ——— | 30 — 27 | rain . ——— . cloudy . |
| 3 | 45 47 45 | $29^{9}$ — — | ——— | 25 15 10 | rain . ——— . ——— . |
| 4 | 44 46 41 | $29^{8}$ — — | ——— | 35 20 — | Rain . . ——— . clear . . |
| 5 | 37 41 42 | $99^{6}$ $29^{5}$ $29^{2}$ | ——— | 24 15 25 | cloudy . . clear . rain in night |
| 6 | 41 43 40 | — $29^{1}$ — | ——— | 30 — 20 | clear . . rain . . clear . . r. in n. |
| 7 | 40 41 — | 29 — — | ——— | 30 25 20 | rain . . cloudy . . rain . r. in n. |
| 8 | 53 — 40 | $29^{1}$ — $29^{4}$ | ——— | 25 — 22 | rain . . ——— . . ——— . |
| 9 | 40 43 41 | $29^{5}$ — $29^{4}$ | ——— | 25 — 26 | clear . ——— . . ——— . . . r. in n. |
| 10 | 45 47 46 | $28^{9}$ $28^{6}$ $28^{8}$ | ——— | 30 25 20 | rain . . . ——— . . . . clear . . halo |
| 11 | 45 46 43 | $29^{2}$ $29^{4}$ — | ——— | 30 25 20 | cloudy . . . . rain . . ——— . r. in n. |
| 12 | 45 46 41 | $29^{5}$ $—^{0}$ 8 | ——— | 95 20 25 | clear . ——— . . rain . clear . . . . |
| 13 | 40 43 41 | 30 $—^{2}$ $—^{1}$ | ——— | 25 17 21 | clear . . . . ——— . . . . ——— . . |
| 14 | 40 42 43 | 30 $29^{8}$ $—^{5}$ | ——— | 20 — 27 | cloudy . . rain . . ——— . . . r. in n. |
| 15 | 51 55 54 | $29^{5}$ — $—^{4}$ | — 10 4 | 15 — — | clear . ——— . . rain . . . . |
| 16 | 53 55 52 | $29^{3}$ — $—^{2}$ | 2 15 10 | ——— | clear . ——— . . rain . . . |
| 17 | 50 51 48 | $29^{3}$ $—^{4}$ — | 10 20 15 | ——— | clear . . ——— . . . ——— . . r. in n. |
| 18 | 47 50 49 | $29^{5}$ $—^{6}$ $—^{5}$ | 19 30 25 | ——— | clear . . . cloudy . . . rain . . . |
| 19 | 50 — 47 | $29^{5}$ $—^{6}$ — | 15 20 — | — — 10 | cloudy . . rain . . ——— . . |
| 20 | 46 47 49 | $29^{6}$ — $—^{5}$ | ——— | 8 10 19 | cloudy . . ——— . . rain . . in n. |
| 21 | 51 55 50 | $29^{5}$ $—^{4}$ $—^{5}$ | ——— | 40 50 43 | rain . . clear . rain . . . |
| 22 | 43 47 49 | $29^{5}$ $—^{7}$ $—^{8}$ | ——— | 30 — 38 | clear . . hail . . . . rain . . |
| 23 | 48 52 49 | $29^{8}$ — — | ——— | 35 25 35 | clear . ——— . . . rain . . |
| 24 | 50 51 48 | $29^{7}$ $—^{6}$ — | ——— | 38 — 40 | fog . . . rain . . . rain . . . |
| 25 | 46 49 45 | $29^{5}$ — — | ——— | 38 39 36 | clear . . rain . . . ——— . . |
| 26 | 45 46 45 | $29^{3}$ $—^{2}$ — | ——— | 38 40 37 | cloudy . . . . rain . . . ——— . . |
| 27 | 43 48 47 | $29^{2}$ $—^{1}$ $—^{0}$ | ——— | 40 — 41 | cloudy . . clear . . cloudy . . |

15. Sensible temperature like Midsummer.
16. ——————— much lowered.

15, 16, 17, 18, and 19. The humidity of the atmosphere, as indicated by the hygrometer, changed from considerable dampness during the early part of the month, to a high degree of dryness; and on the 20th returning to an increased humidity. The hygrometer employed was made by Adams, of the *arista* of the wild oat, and was placed in the open air, but sheltered from rain. The apparent state of the atmosphere gave no indication of this variation in the degree of aqueous solution; nor is there any cause to think that the instrument did not act correctly, as far as its powers go.

22. Stormy showers. Three P. M. heavy fall of hail covering the ground, but soon dissolving: wind S.W. . . . succeeded by a starry evening. On the 30th of October, the approach of winter seen by the first fall of snow.

*Pince's-Street, Cavendish-Square, Nov. 29, 1810.*

* The degree of any state of the weather is indicated by a number of points added to the description. Thus *clear* . is the first approach to a bright atmosphere; *clear* . . . . . indicates its most unclouded state.

